# Expiratory Peak Flow and Minute Ventilation Are Significantly Increased at High Altitude versus Simulated Altitude in Normobaria

**DOI:** 10.3390/life12020306

**Published:** 2022-02-17

**Authors:** Nikolaus C. Netzer, Linda K. Rausch, Matthias Frieß, Kingman P. Strohl, Robert Schilz, Michael Decker, Stephan Pramsohler

**Affiliations:** 1 Hermann Buhl Institute for Hypoxia and Sleep Medicine Research, Institute of Sport Science, University Innsbruck, 83043 Bad Aibling, Germany; linda.rausch@uibk.ac.at (L.K.R.); m.friess@uibk.ac.at (M.F.); s.pramsohler@gmx.net (S.P.); 2 Institute of Mountain Emergency Medicine, Eurac Research, 39100 Bozen, Italy; 3 Department of Medicine, Division of Sport Medicine and Rehabilitation, University Hospitals, 89070 Ulm, Germany; 4 Department of Pulmonary and Critical Care Medicine, University Hospitals, Case Western Reserve University, Cleveland, OH 44106, USA; kpstrohl@aol.com (K.P.S.); r.schilz@cw.com (R.S.); 5 Department of Physiology, Institute for Aerospace Physiology, Case Western Reserve University, Cleveland, OH 44106, USA; michael.decker@case.edu

**Keywords:** spiroergometry, altitude, air density, normobaria, hypobaria, ventilation, exercise

## Abstract

Simulated altitude (normobaric hypoxia, NH) is used to study physiologic hypoxia responses of altitude. However, several publications show differences in physiological responses between NH and hypobaric conditions at altitude (hypobaric hypoxia, HH). The causality for these differences is controversially discussed. One theory is that the lower air density and environmental pressure in HH compared to NH lead to lower alveolar pressure and therefore lower oxygen diffusion in the lung. We hypothesized that, if this theory is correct, due to physical laws (Hagen-Poiseuille, Boyle), resistance respectively air compression (Boyle) at expiration should be lower, expiratory flow higher, and therefore peak flow and maximum expiratory flow (MEF) 75–50 increased in hypobaric hypoxia (HH) vs. normobaric hypoxia (NH). To prove the hypothesis of differences in respiratory flow as a result of lower alveolar pressure between HH and NH, we performed spirography in NH at different simulated altitudes and the corresponding altitudes in HH. In a cross over study, 6 healthy subjects (2 f/4 m, 28.3 ± 8.2 years, BMI: 23.2 ± 1.9) performed spirography as part of spiroergometry in a normobaric hypoxic room at a simulated altitude of 2800 m and after a seven-hour hike on a treadmill (average incline 14%, average walking speed 1.6 km/h) to the simulated summit of Mauna Kea at 4200 m. After a two-month washout, we repeated the spirometry in HH on the start and top of the Mauna Kea hiking trail, HI/USA. Comparison of NH (simulated 4200 m) and HH at 4200 m resulted in increased pulmonary ventilation during exercise (VE) (11.5%, *p* < 0.01), breathing-frequency (7.8%, *p* < 0.01), peak expiratory flow PEF (13.4%, *p* = 0.028), and MEF50 (15.9%, *p* = 0.028) in HH compared to NH, whereas VO_2max_ decreased by 2%. At 2800 m, differences were only trendy, and at no altitude were differences in volume parameters. Spirography expresses higher mid expiratory flows and peak flows in HH vs. NH. This supports the theory of lower alveolar and small airway pressure due to a lower air density resulting in a lower resistance.

## 1. Introduction

The use of simulated altitude (Normobaric Hypoxia, NH) has become increasingly popular in the past years due to a rising number of commercially available devices (e.g., facemasks, tents, rooms) with increased nitrogen volume percentage and reduced oxygen volume percentage, claiming a similar effect to being exposed to terrestrial altitude or lowered environmental pressure in a hypobaric chamber, i.e., hypobaria (Hypobaric Hypoxia, HH). However, when testing humans, completing exercise tasks or resting in NH and HH, results on ventilatory parameters are not consistent between the two environments. Basualto-Alarcon and colleagues (2012) found enhanced cardiorespiratory parameters as well as lower oxygen saturation levels in HH compared to NH during exposure to 3000 m and aerobic exercise performance [1]. A meta-analysis by Coppel et al. (2015) lists further studies presenting such differences between NH and HH [2].

The underlying physiological mechanism could result in a higher respiratory exchange ratio (RER) in HH, which is attributed to enhanced respiratory work due to higher gas density and gas flow rate in NH [3]. Richard and Koehle (2012) suppose that a higher gas flow rate in NH causes elevated airway resistance, which in turn leads to enhanced respiratory work compared to HH [4]. On the contrary, Faiss and colleagues (2013) found only slightly lower minute ventilation and higher tidal volumes in HH compared to NH when performing submaximal exercise tests while being exposed to 3000 m for 24 h. In addition, they reported no changes in breathing frequency or oxygen saturations [5].

Theoretically, the lower air density at altitude should improve airflow dynamics. Finkelstein et al. (1965) exposed 10 patients with COPD and a mean FEV1/forced vital capacity (FVC) ratio of 51% to the equivalent of 5488 m in a hypobaric chamber and found that the vital capacity fell from a mean of 2.97 L to 2.72 L while the FEV1/FVC ratio improved, increasing from 51 to 57%. They also noted improvement in MVV from 60 to 73 L/min and improvements in the maximal expiratory flow rates from 1.45 to 1.55 L/s [6]. In opposition to these findings, in their study of 18 COPD patients with a mean baseline FEV1 of 31% predicted, Dillard et al. (1998) found no statistically significant differences in vital capacity, FEV1, MVV, or PEFR at a simulated altitude of 2348 m [7]. 

Data on airway resistance, which is reflected in parameters of spirometry, is still scarce comparing NH to HH. Most studies investigate only one of the two conditions without comparing both. A significant decrease was reported in expiratory flow-volume parameters in HH concentrating on volume parameters, such as FVC and FEV1 in climbers during ascents to high altitudes [8]. Cao et al. (2019) also found expiratory volume limitation in runners performing maximal endurance tests [9]. Other respiratory parameters did not differ significantly. In opposition to these described decreases in airflow, several authors in the past have reported increased expiratory flows [10,11,12]. The problem with previous publications on lung function at altitude is the difficult comparability because the circumstances differ in altitudes and acclimatization times, and several publications compare lung function tests of sea level measurements to states of suspected pulmonary edema. 

We hypothesize that there is a difference in airway resistance between NH and HH due to reduced air density with lower environmental and small airway as well as alveolar pressure in HH because the airways in the lung are an open-communicating system with the environment. Consequently, we hypothesize that lung function parameters which express airway resistance closely, such as peak flow and middle expiratory flows (MEF), are different between HH and NH. The lower alveolar and small airway pressure subsequently could lead to the above described lower oxygen saturation due to lower oxygen diffusion with all its cardiovascular consequences. 

Since former studies in the 20th century regarding changes in airflow at altitude as mentioned above only studied COPD and pulmonary edema patients and only compared HH to sea-level, to prove our hypothesis, we performed submaximal ergospirometry including spirography at 2800 m and 4200 m simulated (NH) altitude and at a mountain (HH) in a cross over design to assess resistance-related flow parameters. 

## 2. Materials and Methods

### 2.1. Study Design

The actual study is designed as an interventional cross-over trial. 

### 2.2. Subjects

A total of 6 healthy students from the University of Innsbruck (Austria) were recruited via blackboard and social media announcement for the study (4 men and 2 women, aged 24 to 45 years). Volunteers underwent a routine medical examination to ensure their ability to participate in the study. Exclusion criteria were chronic or acute cardiovascular or respiratory diseases, smoking, chronic headache or migraine, and neurological or psychological diseases. Each participant reported a habitual residence below 1500 m of terrestrial altitude. None of the participants were exposed to >2000 m of terrestrial or artificial altitude for 6 months prior to the start of the study. Additionally, participants reported being physically active at a moderate level of intensity for more than 150 min per week. Participants were informed about the two test trials of the study procedure as well as about potential risks and gave written informed consent prior to the study’s start. The Ethics Committee at the Leopold-Franzens-University of Innsbruck, Austria, approved the study protocol.

### 2.3. Study Protocol

Participants performed four submaximal exercise tests on a treadmill with a standardized procedure (described below) in two different hypoxic trials (T1 and T2). T1 was conducted in simulated altitude (NH) in Bad Aibling (Germany, 450 m). The chamber had a dimension of 102 m^2^ with a nitrogen expulsion system (Low Oxygen Systems Inc., Berlin-Buch, Germany) that provided a mixture of fresh air in order to control CO_2_ levels comparable to the levels measured in hypobaria (HH). Four weeks after the first trial, participants underwent an equivalent testing protocol in HH at Mauna Kea Mountain, HI, USA. Mauna Kea was chosen for a number of crucial reasons. The mountain’s altitude with a moderate continuous inclination allowed the simulated treadmill hike to be programmed to a high degree of comparability. Constant climatic conditions on the mountain with temperatures around 20 °C paralleled the temperatures in the simulated altitude (NH) room. Road access to the summit facilitated the transport of gear and descent of the participants. Participants were asked to wear the same clothes and use the same equipment during each trial. The amount of food and beverage intake was weighed and recorded for each person at breakfast and dinner before T1. Volunteers were instructed to follow the same food and beverage intake in T2. Participants carried the same weight consisting of snacks and beverages in a backpack during both trials. Energy and fluid intake were measured individually during T1 and kept the same for T2. Temperature and humidity were continually measured in both settings.

### 2.4. Submaximal Exercise Testing

In T1, spiroergometry was performed on a treadmill (h/p Cosmos Mercury and Quasar). The built-in calibrating sensor of the treadmill was checked for accuracy by a mechanic prior to testing (error < 0.2 m per 100 m interval). Before each test, participants had a five-minute resting period. Tests were carried out at 2800 m and 4200 m of simulated altitude. Participants completed a distance of 500 m with an average incline of 14.2% on the treadmill. The incline of the treadmill was determined as the ratio between the difference in altitude (1405 m) and the walking distance (9894 m) from the start of the Humu’ula trail at 2800 m until the top of Mauna Kea (4205 m). Participants were instructed to keep 80% of maximal heart rate throughout the test duration. Maximal heart rate was calculated by applying the heart rate formula of Hollman and Hettinger (2000). Walking speed was individually adjusted according to heart rate references. Heart rate (chest belt, Polar, Finland), arterial oxygen saturation, ventilatory, and gas exchange parameters were continuously measured throughout the test (Oxycon Mobile, former Care Fusion, now Vyaire, Hoechstaedt, Germany and Chicago, IL, USA). There was a 6 h period between the 2 measurements, where participants performed moderate-intensity physical activity on treadmills with an average incline of 14.2% and an average speed of 1.6 km/h in order to simulate the hike on top of Mauna Kea. The treadmill speed was calculated as a function of the duration of walking (6 h) and the walking distance. Inspired fraction of O_2_ in the chamber was modified automatically, which led to the completion of the treadmill task at a simulated altitude of 4200 m. The following parameters were assessed via spirometry: average aerobic capacity (VO_2_), average minute ventilation (VE), and breathing frequency (BF). The first four averaged breathing rates were not statistically assessed in order to avoid bias due to individually different adaption times for fixed heart rate frequencies. 

In T2, spiroergometry was equivalently performed in terrestrial altitude at 2500 m and 4200 m on the mountain site of Mauna Kea, Big Island, HI, USA. Spiroergometry was carried out on a 500 m concrete road with an average incline of 14.2%. Participants were transported by car to the starting point of the first spiroergometry at 2800 m. The second measurement point at 4200 m they reached by foot after a 6 h hike with an average speed of 1.6 km/h (GPS watch, Garmin, Forerunner, Switzerland). The hike was monitored by a physician, and support staff by car was available. 

Prior to every spiroergometry test in both conditions, T1 and T2, participants performed a lung function test with the same measuring device in order to determine forced vital capacity (FVC), forced expiratory volume in the first second (FEV1), peak expiratory flow (PEF), as well as maximal expiratory flow at 75% and 25% of vital capacity (MEF). The device was volume and flow calibrated before each of the four measurement points in T1 and T2. In order to avoid the major influence of increased respiratory workload at HH compared to NH, there was a 20-min resting break after exercise in both NH and HH before spirometry at simulated and real 4200 m altitude. 

### 2.5. Statistics

Although the sample size was primarily based on logistical considerations, a power analysis was performed with G-Power Version 3.1.9.2 (University of Kiel) for dependent samples (correlation coefficient set at 0.5) and a power of 80% with expected values of mean peak expiratory flow of 7.5 L/s in NH (according to EU EN 13826 for a 25 year old, 175 cm tall) and an expected mean peak expiratory flow of 9.2 L/s in HH with a standard deviation of 1 L/s. The power analysis yielded a sample size of six subjects. Further statistical analysis was performed using IBM SPSS Statistics 23 (PASW Statistics for Windows version 21.0, SPSS Inc., Chicago, IL, USA). Data were expressed as means and standard deviations or as medians with 95% confidence intervals as appropriate. Nonparametric tests (Wilcoxon) were used for peak expiratory flow (PEF), forced expiratory volume in the first second (FEV1), mean expiratory flow 25–75 (MEF 25–75), forced vital capacity (FVC), and minute ventilation (VE), oxygen uptake (VO_2_) comparing NH and HH. The significance threshold was set at 0.05.

## 3. Results

Subjects’ characteristics, as well as ambient conditions in NH and HH (fraction of inspired oxygen, barometric pressure, temperature), are portrayed in Netzer et al. (2017) [13]. This mentioned work also displays heart rate and oxygen saturation values in NH and HH during the (simulated) hike from 2800 m to 4200 m.

**Figure 1:**
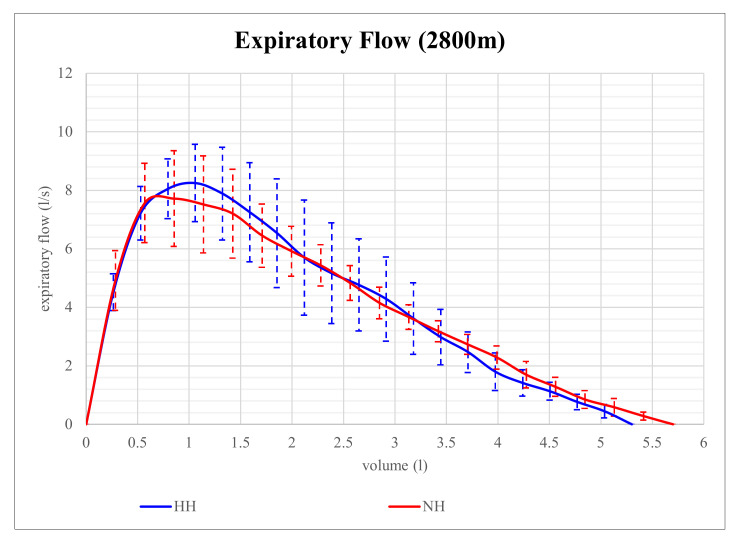
Mean expiratory flow volume in 2800 m of normobaric hypoxia (NH) and hypobaric hypoxia (HH). Residual Volume (RV) can be calculated via space under the curve. The mean parameters of each group are displayed. The error bars represent SEMs.

**Figure 2:**
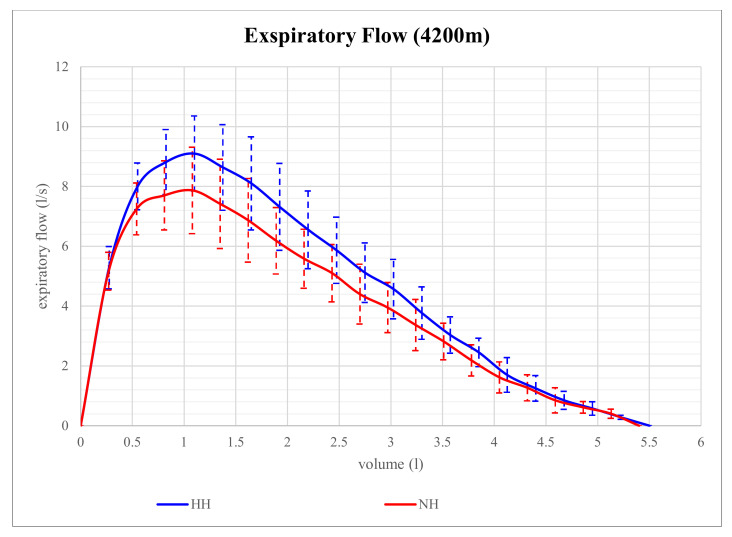
Mean expiratory flow volume in 4200 m of normobaric hypoxia (NH) and hypobaric hypoxia (HH). Residual Volume (RV) can be calculated via space under the curve. The mean parameters of each group are displayed. The error bars represent SEMs.

Expiratory flow volume in NH and HH at 2800 m is shown in Figure 1. Although expiratory flow volume seems to be higher in HH than in NH at that level of altitude, the differences in parameters of expiratory flow between NH and HH were not significant. However, at 4200 m, expiratory flow volume proved to be significantly different and is shown in Figure 2. At that altitude level, significant differences between NH and HH were found in PEF, MEF75, and MEF50. PEF was 13.4% higher in HH than in NH (*p* = 0.028), MEF75 was 16.2% higher in HH than in NH (*p* = 0.028), and MEF50 was 15.9% higher in HH than in NH (*p* = 0.046). No significant differences between NH and HH were found in FEV1 at 2800 m (*p* = 0.496) as well as at 4200 m (*p* = 0.249). FVC did also not show any significant differences between NH and HH at 2800 m (*p* = 0.173) as well as at 4200 m (*p* = 0.6).

**Figure 3:**
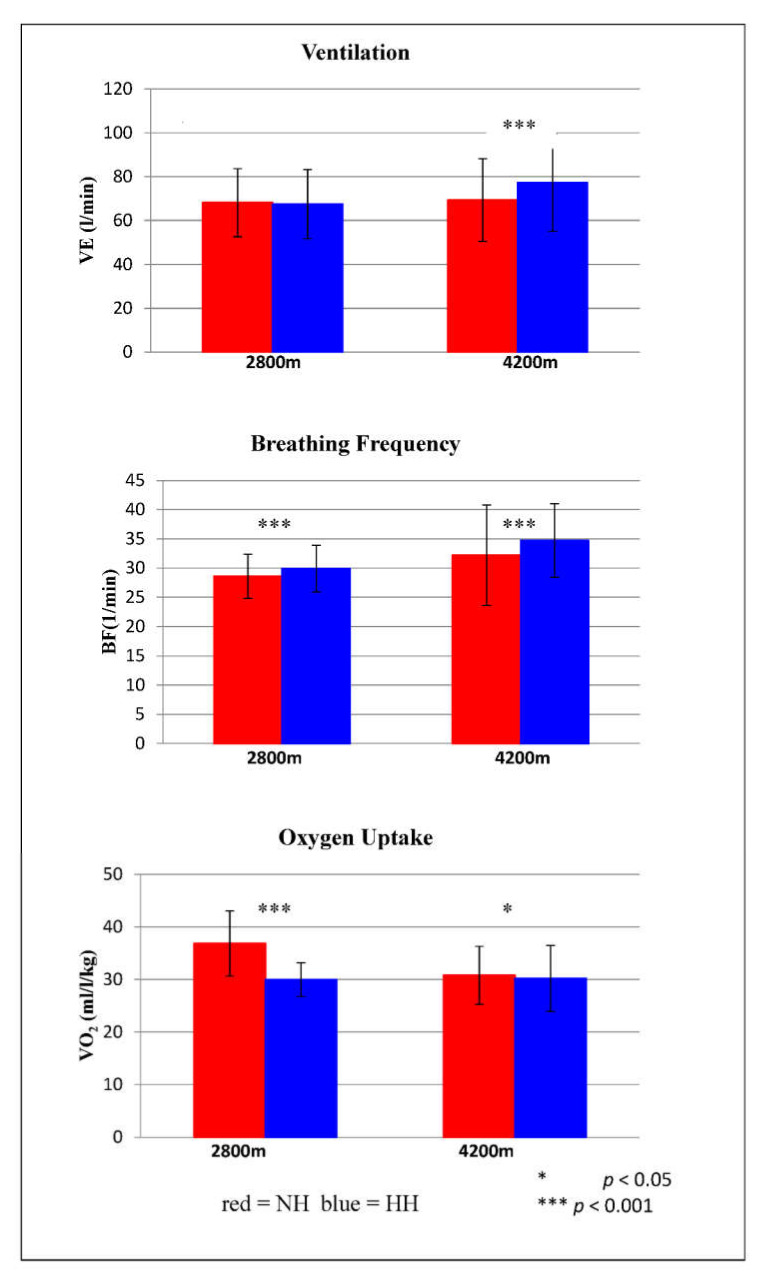
Mean ventilation (VE), breathing frequency (BF), and oxygen uptake (VO_2_) in 2800 m and 4200 m normobaric (NH) and hypobaric hypoxia (HH) during submaximal spiroergometry. The mean parameters of each group are displayed. The error bars represent SEMs.

Ventilatory parameters during submaximal exercise testing are shown in Figure 3. VE differed significantly at 4200 m (*p* < 0.001). On average, VE was 11.5% higher in HH than in NH. BF showed significant differences at 2800 m (*p* < 0.001) and at 4200 m (*p* = 0.001). At 2800 m, BF was on average 4.3% lower in NH than in HH, and at 4200 m, BF was on average 7.8% higher in HH than in NH. VO_2_ also differed significantly at both altitude levels. At 2800 m, VO_2_ was on average 23% higher in NH than in HH (*p* < 0.001), and at 4200 m, VO_2_ was on average 2% higher in NH than in HH (*p* = 0.029).

## 4. Discussion

To the best of our knowledge, this is the first study comparing parameters of especially small airway resistance-related expiratory flow parameters with spirography within ergospirometry during submaximal exercise in normobaric and hypobaric conditions at two different altitude levels in the same cohort with a cross-over study design.

Our spirometric results showed that mid expiratory flow and peak flow are significantly different between NH and HH regarding an altitude of 4200 m. This finding confirms the assumption of Loeppky and colleagues (1997), as well as Richard and Koehle (2012), that an increased airway resistance due to a higher gas density in NH causes increased ventilatory work [3,4]. It is also consistent with the early presentation of Finkelstein et al. (1965) of increased FEV/FVC ratio in COPD patients at high altitudes in assumption of reduced air density at high altitudes [6]. Cross et al. (2018) discuss in their recent publication that flow volume measured at the mouth might be underestimated at sea level due to the higher thoracic gas compression and should be corrected by a method published by Pedersen and Ingram (1987), which involves the difference of barometric pressures with Boyle’s law [14,15]. Cross and colleagues measured flow volume curves at Mount Kilimanjaro at different altitudes (853 m and 4837 m) and found increased MEF’s and peak flow at altitude after adjusting for thoracic gas compression [14]. When they further adjusted for air density, the flows were reduced at high altitude compared to 853 m. This would support our findings in another study with healthy adults.

At lower altitudes, namely 2800 m, differences between NH and HH were less pronounced in our study but still visible. This could be attributed to the short exposure to hypobaria when we measured on Mauna Kea at 2800 m compared to a prolonged exposure time to hypobaria with the hike in between when we measured at 4200 m. Debevec and colleagues (2014) also support the hypothesis that differences in respiratory parameters as well as in peripheral capillary oxygen saturation (SpO_2_) between HH and NH only appear after more than six hours [16].

Our results, showing a higher peak flow at hypobaric hypoxia, support our hypothesis that a lower environmental pressure could lead to a lower air and oxygen partial pressure in the lung downwards to the small airways and alveoli (PAO_2_) and therefore based on Hagen-Poiseuille’s law there should be lower airway resistance at expiration. Conkin et al. (2016) discussed such a hypothesis already based on the assumption that inspired oxygen partial pressure (PiO_2_) is influenced by altitude (FiO_2_ < 0.21) using the equation PiO_2_= (PB-47) × FiO_2_ thus influencing the alveolar gas equation PAO_2_ = PiO_2_ = (PB-47) × FiO_2_ − PACo_2_ × [FiO_2_ + ((1 − FiO_2_)/RER)] (RER = Respiratory Exchange Rate). However, this has not been proven in a scientific experiment so far [17].

A limitation of our study is that we were not able, due to logistic restrictions on-site at Mauna Kea, to measure lung diffusion capacity. Therefore, we cannot prove or assume that a lower PAO2 would really lead to lower oxygen diffusion and explain the in several papers, including our previous publication, described lower oxygen saturation at HH vs. NH.

As a smaller limitation for the study, we regard the differences in ambient factors. We could not exclude ultraviolet radiation from the sun in the field condition, which could have led to increased fluid loss and consequently more dehydration on the mountain site in HH compared to chamber conditions in NH, thus influencing airway mucosa hydration and following differences in airway resistance. An equal amount of liquid was consumed during the time of exposure in both hypoxic conditions in order to maintain comparability. We could keep ambient temperatures at a comparable level under both conditions, around 21 °C. This was one main reason why we have chosen Mauna Kea as a mountain site with its comfortable temperature conditions during the daytime.

Since volume parameters FVC and FEV1 at both altitude levels were not significantly different in both conditions, and this has been shown before by others [5], we doubt that the amount of single breath inspired air has a larger influence on the difference in oxygen saturation levels under short exposure conditions. In contrast, Sharma and Brown (2007) report a significant decline of FVC due to a 3-day hypobaric hypoxia exposure to 3450 m, whereas, after acute hypoxic exposure, FVC increases initially [18]. As responsible mechanisms, the authors suppose that exhaustion of respiratory muscles or a higher lung blood volume causes the delayed decline in FVC [8]. The lacking decrease in FVC in both hypoxic conditions of our study indicates that exposure time was not long enough to cause exhaustion of respiratory muscles.

Whereas single breath volumes showed no difference, submaximal exercise tests showed a significant increase in VE at 4200 m in HH vs. NH. Via increased minute ventilation through increased breathing frequency, the body seems to compensate for a possible lower diffusion capacity. In contrast to this finding, Coppel et al. (2015) summarized five studies exhibiting a decrease in minute ventilation in HH compared to NH, and only two studies did not show significant effects between conditions [2]. For example, Savourey and colleagues (2003) support the increased findings in HH, stating that a decrease in air density is related to a decrease in tidal volume, which could serve as an explanation for the higher VE in HH [19]. Thereby connected is a higher BF in order to counteract the decrease in tidal volume [19]. This explanation of underlying physiological mechanisms supports the outcome of our study, which is reflected by the measurements of Ogawa et al. (2019) with measurements in an environmental chamber with hypobaric and normobaric hypoxia [20]. Moreover, Bastualto-Alarcon et al. (2012) conducted similar submaximal exercise tests at 3000 m of normobaric and hypobaric hypoxic exposure [1]. They also found elevated minute ventilations in HH and argued that sympathetic and vagal modulations could be responsible for this outcome.

Our results, in combination with previous findings of other authors, are clinically and physiologically relevant because more and more tests and acclimatization preparations in occupational medicine and for professional and recreational mountaineers are done in normobaric hypoxia since it is less costly than acclimatization on-site or in hypobaric chambers. However, the differences between HH and NH have to be taken into consideration using normobaric hypoxia for these interventions.

In conclusion, expiratory flow at the beginning of expiration, peak flow, and MEF 75–50 is higher at high altitude vs. simulated high altitude under normobaric conditions. This could be an expression of lower alveolar and small airway air pressure. Future studies measuring lung diffusion capacity and maybe imaging techniques in larger climatized hypobaric chambers should give further insights into this clinically relevant phenomenon.

## Data Availability

Data can be shared if requested.
